# Robotic Bronchial Restapling After Inadvertent Stapling of an Endobronchial Suction Tube: Technical Note and a Prestapling Time-Out

**DOI:** 10.1093/icvts/ivag261

**Published:** 2026-09-17

**Authors:** Sang Yun Song, Kyo Seon Lee, Kook Joo Na, Ju Sik Yun

**Affiliations:** Department of Thoracic and Cardiovascular Surgery, Chonnam National University Hwasun Hospital, Chonnam National University Medical School, Hwasun-eup, Jeollanam-do 58128, Republic of Korea; Department of Thoracic and Cardiovascular Surgery, Chonnam National University Hospital, Chonnam National University Medical School, Gwangju 61469, Republic of Korea; Department of Thoracic and Cardiovascular Surgery, Chonnam National University Hwasun Hospital, Chonnam National University Medical School, Hwasun-eup, Jeollanam-do 58128, Republic of Korea; Department of Thoracic and Cardiovascular Surgery, Chonnam National University Hwasun Hospital, Chonnam National University Medical School, Hwasun-eup, Jeollanam-do 58128, Republic of Korea

**Keywords:** robotic surgery, lobectomy, bronchus, intraoperative complication

## Abstract

Foreign body entrapment during bronchial stapling is rare but may result in severe airway injury. We report an unusual intraoperative complication during robot-assisted thoracic surgery (RATS) lobectomy: inadvertent stapling of an endobronchial suction tube into the right lower lobe (RLL) bronchus, successfully managed robotically without conversion. A 65-year-old woman with clinical stage I lung adenocarcinoma underwent RATS RLL lobectomy with mediastinal lymph node dissection. During bronchial division, an endobronchial suction tube used to facilitate lung deflation was incorporated into the staple line. The tube was removed through limited resection of the involved bronchial stump segment, followed by traction-suture-assisted restapling. The postoperative course was uneventful, and 1-year follow-up showed no atelectasis and a patent right middle lobe bronchus with mild narrowing. When an adequate bronchial margin remains, robotic bronchial restapling may provide a feasible rescue option without conversion. A focused “pre-stapling time-out” to confirm the removal of endobronchial adjuncts may help prevent similar events.

## INTRODUCTION

Robot-assisted anatomical lung resection is increasingly used for early-stage lung cancer, with favourable perioperative outcomes and low conversion rates reported in selected settings.^1^ As adoption of this approach grows, the recognition and management of uncommon intraoperative complications become increasingly important. Bronchial stapling is generally reliable; however, inadvertent inclusion of a foreign body within the bronchial lumen, although rare, may result in severe airway injury.

We describe the entrapment of an endobronchial suction tube during stapled division of the right lower lobe (RLL) bronchus in robot-assisted thoracic surgery (RATS) lobectomy and its successful management by limited bronchial stump resection and traction-suture-assisted restapling, without conversion.

## CASE PRESENTATION

A 65-year-old woman with clinical stage I lung adenocarcinoma underwent RATS RLL lobectomy with mediastinal lymph node dissection. The procedure was performed using the da Vinci Xi system (Intuitive Surgical) through 4 ports, including a utility incision, under one-lung ventilation with carbon dioxide insufflation. An endobronchial suction tube was inserted to facilitate lung deflation.

The operation was uneventful until bronchial division. After stapled transection of the RLL bronchus, inspection of the stump revealed that the suction tube had been incorporated into the staple line (**Figure 1A**). The tube was not forcibly removed to avoid shear injury to the bronchus. Although conversion for bronchial repair was considered, the remaining bronchial stump had a sufficient margin for robotic salvage. The involved stump segment was excised en bloc with the entrapped tube (**Figure 1B**). Prolene traction sutures were placed to optimize alignment and apposition of the bronchial edges (**Figure 1C**), and the bronchus was restapled under countertraction to create a new RLL bronchial stump (**Figure 1D and E**). Adequate right middle lobe (RML) expansion was confirmed (**Figure 1F**). No conversion was performed, and no adjunctive reinforcement material was used.

**Figure 1.**
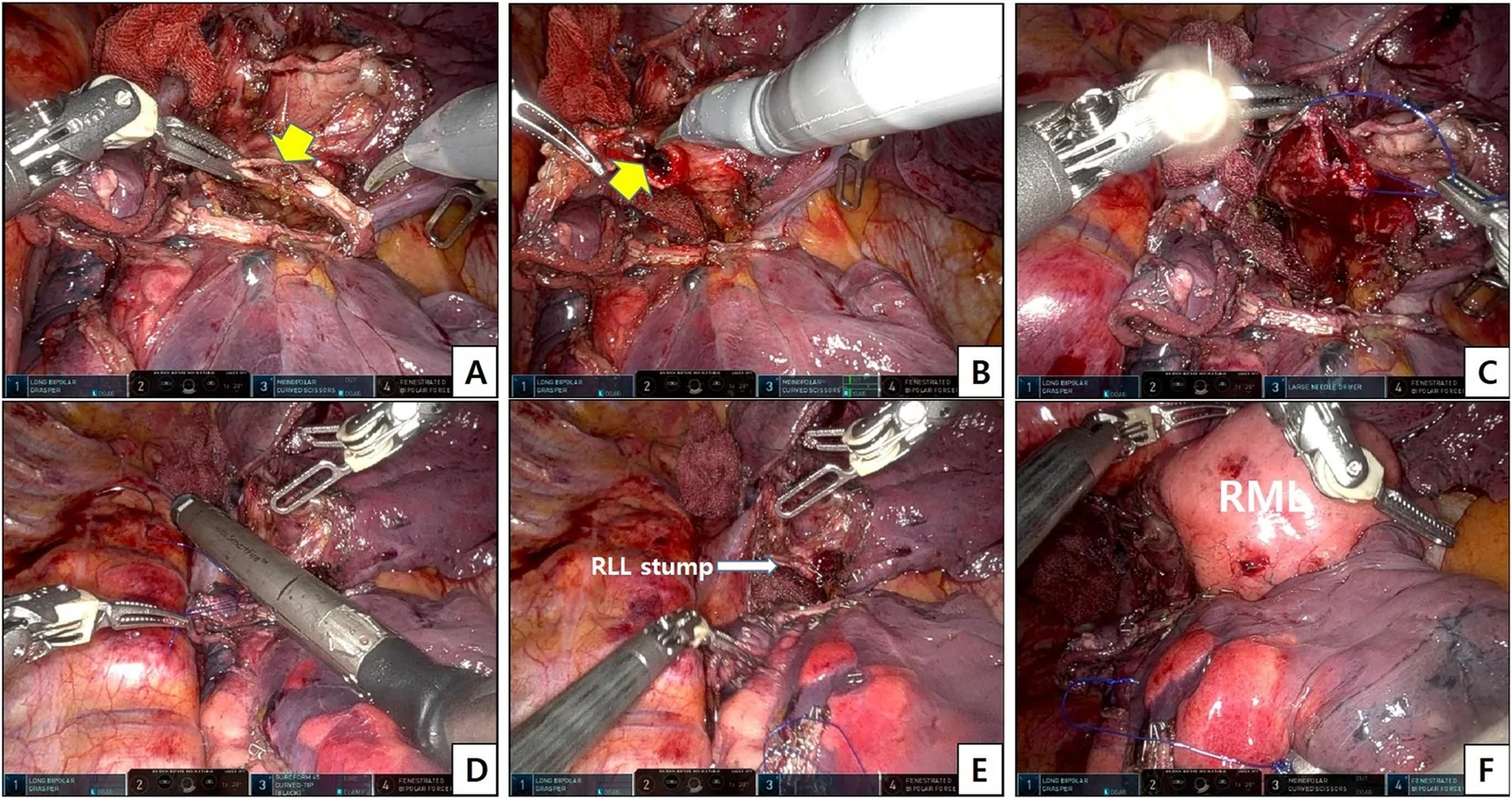
Intraoperative Findings. (A) Suction tube (arrow) entrapped in the stapled RLL bronchus. (B) En bloc excision of the involved stump segment with the suction tube (arrow). (C) Traction sutures. (D) Restapling of the RLL bronchial stump. (E) Newly created RLL bronchial stump. (F) Right middle lobe expansion after bronchial reclosure

The postoperative course was uneventful. At the 1-year follow-up, chest radiography showed no atelectasis (**Figure 2A**), and computed tomography (CT) revealed a patent RML bronchus with mild narrowing (**Figure 2B and C**). The patient reported no respiratory symptoms, including exertional dyspnoea, and pulmonary function testing was unremarkable.

**Figure 2.**
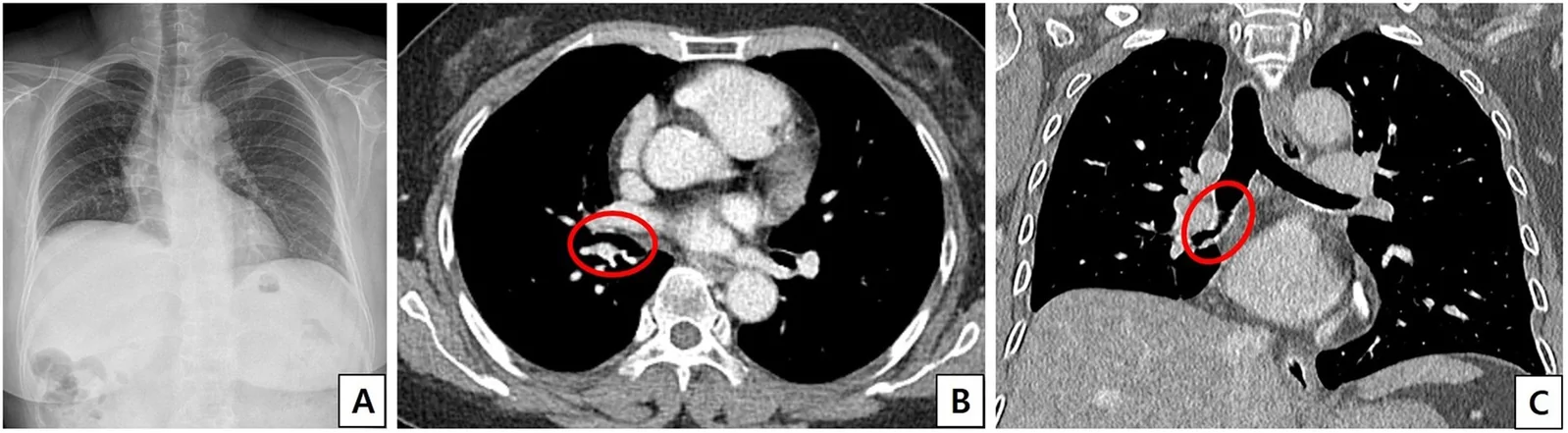
Postoperative Follow-Up Images at 1 Year. (A) Chest radiograph. (B and C) Chest CT: axial (B) and coronal (C) views; the red circle indicates the RML bronchus

## DISCUSSION

Inadvertent stapling of an endobronchial suction tube during bronchial division is exceptionally rare but may cause bronchial disruption, prolonged air leak, infection, or airway stenosis if luminal geometry is compromised. Tanaka et al^2^ reported a similar event during robot-assisted right lower lobectomy that was managed by staple removal and bronchial stump resuturing, with coverage using a pedicled pericardial fat pad. In the present case, the complication was also managed without conversion; bronchial restapling was performed because sufficient stump margin remained after limited resection. Bronchial restapling may be considered only when an additional division can be performed without jeopardizing the remaining bronchus, creating excessive tension, or leaving an excessively short or distorted stump. If the margin is inadequate or deeper bronchial injury is suspected, formal bronchial repair should remain a low-threshold option. Although buttressing with a fat pad or pleural flap may be appropriate in selected situations, such as friable tissue, inflammation, steroid exposure, or prior chemoradiotherapy, reinforcement was unnecessary in this case because the staple line was well aligned and tension-free, and tissue quality and bronchial margin were adequate.

The feasibility of “robotic rescue” is consistent with broader reports on management of serious intraoperative complications during RATS. Takase et al^3^ and Chauhan et al^4^ reported that selected bronchial injuries could be addressed with robotic primary repair, emphasizing that, with appropriate expertise and case selection, complex intraoperative troubleshooting can be completed within the robotic platform. Nevertheless, robotic rescue should be regarded as an option rather than a requirement, and used only when patient safety can be maintained.

When airway adjuncts are used, the risk involves a shared anaesthetic–surgical field. The World Health Organization time-out concept emphasizes team communication and structured checks to improve safety and compliance.^5^ We propose a targeted pre-stapling time-out immediately before bronchial stapling to confirm (1) the presence and position of any endobronchial device, (2) withdrawal or removal, and (3) explicit verbal confirmation between the anaesthesiologist and surgeon that the bronchial lumen has been cleared. This check is brief and reproducible. More importantly, strictly limiting the duration that any device remains within the bronchial lumen and adopting a protocol for immediate removal after use may provide a more effective and robust safeguard against shared-field entrapment.

Foreign body entrapment during bronchial stapling in RATS lobectomy may be managed robotically in selected cases. Here, limited bronchial resection, extraction, traction-suture-assisted alignment, and restapling achieved a favourable outcome without conversion. A brief pre-stapling time-out, together with minimizing endobronchial device dwell time, may help prevent similar events.

## Data Availability

The data underlying this article are available in the article.
